# Use of Epidural Analgesia in Children With Neuromuscular Conditions Following Hip Reconstruction

**DOI:** 10.7759/cureus.30522

**Published:** 2022-10-20

**Authors:** Sean Tabaie, Aribah Shah, Omar Tarawneh, Grace Blaylock, Evan Sheppard, Kevin Cho

**Affiliations:** 1 Orthopaedic Surgery, Children's National Hospital, Washington D.C., USA; 2 Orthopaedic Surgery, George Washington University School of Medicine and Health Sciences, Washington D.C., USA; 3 School of Medicine, New York Medical College, Westchester, USA; 4 Orthopaedic Surgery, Children's Hospital of Philadelphia, Philadelphia, USA; 5 Sheikh Zayed Institute for Pediatric Surgical Innovation, Children's National Health System, Washington D.C., USA

**Keywords:** complications, postoperative pain, cerebral palsy, hip surgery, epidural analgesia

## Abstract

Background: Neuromuscular conditions, such as cerebral palsy, are the most common motor disabilities in the pediatric population. Children with these conditions frequently have accompanying hip deformities that require pelvic and femur osteotomy to correct the spastic hip dislocations. However, postoperative pain management remains an elusive and challenging problem. The purpose of this study was to determine whether postoperative use of epidural analgesia in patients with neuromuscular conditions provided similar outcomes with regard to pain scores, length of stay, duration of foley placement, duration of pain control, and complications as compared to traditional pain management regimens. To our knowledge, this is the first study comparing the use of epidural analgesia to conventional pain relief modalities following hip reconstruction in patients with neuromuscular conditions.

Methods: A retrospective cohort study was performed using records of pediatric patients with neuromuscular conditions treated at our tertiary care center between January 2009 and December 2019. Patients with neuromuscular conditions treated with epidural or non-epidural analgesia for pain relief following unilateral or bilateral proximal femoral osteotomies, pelvic osteotomies, or open hip reduction were eligible for study inclusion. Multiple linear regression was used to determine differences in length of stay, pain score, pain modality, duration of Foley placement, and complications between the two cohorts.

Results: Seventy patients met the inclusion criteria for the study. In all, 58 patients underwent unilateral procedures, of which 30 (52%) received epidural analgesia, and 28 (48%) received non-epidural pain control modalities. Demographic and baseline characteristics were similar among the cohort, except for BMI, which varied slightly. Average pain scores and pain control duration were not statistically different between the pain control modalities. After controlling for demographics, procedure, and immobilization type, the epidural group experienced significantly increased length of stay (+3.18 days, P=0.032) and duration of Foley placement (+1.04 days, P=.013). Complication rates between the two groups were not statistically significant.

Conclusions: The use of epidural analgesia in children with neuromuscular conditions was associated with comparable pain scores, despite the increased length of stay and duration of Foley placement. No statistically significant difference in complication rates was observed between patients receiving epidural anesthesia and those receiving traditional pain modalities.

## Introduction

Neuromuscular conditions, such as cerebral palsy (CP), represent a heterogeneous group of permanent motor disorders that appear early in life. Prevalence rates suggest CP occurs at a rate ranging from 1 to 4 per 1000 births making it the most common childhood motor disorder. Although the etiology remains unclear, CP is an upper motor neuron disease that results from insult to the brain during early development [1]. In addition to the potential for intellectual disability, affected children frequently experience a variety of musculoskeletal complications, including hip subluxation and displacement as a result of asymmetric muscle imbalances, and deformity of the femoral head and acetabulum [2].

Decreased range of motion, pain, and joint contractures are also characteristic of CP, with one of the most common locations being the hip. Depending on the severity, musculoskeletal manifestations can result in modest impairment or severe disability affecting the patient's posture, gait, and participation in activities [3]. Moreover, incidence of hip dislocation directly correlates with Gross Motor Function Classification System (GMFCS) level [4]. The musculoskeletal manifestations of cerebral palsy therefore pose a tremendous challenge for the orthopaedic surgeon. Given the lack of curative therapy, much of the lifelong treatment emphasizes reducing pain and improving the child’s quality of life. Non-operative treatment includes physical therapy, antispasmodics such as botulinum toxin, and bracing. When operative correction is indicated, proximal femur osteotomy and pelvic osteotomy can be performed to relieve joint contractures and correct spastic hip dislocations.

Post-surgical treatment of pain, in these often cognitively impaired children, also represents a significant challenge. Most of these patients suffer from severe intellectual delay and often have issues with communication and, therefore, cannot self-report their pain [5-7]. Since most current forms of pain assessment rely on self or parent reporting, these patients are at risk for being inadequately treated for their pain. While undertreated pain leads to patient suffering, overtreatment can lead to respiratory compromise in an already vulnerable population [5,7-8], along with prolonged hospital stays [8-9]. In patients with neuromuscular conditions undergoing spine surgery, epidural analgesia has been shown to decrease intensive care unit admissions due to respiratory decompensation [8-9]. Specifically, Moore et al. showed that epidural analgesia significantly improved pain control and decreased respiratory depression [8]. To mitigate the respiratory complications associated with scheduled opioid use in this population, many centers have begun using epidural anesthesia in addition to narcotics for children undergoing lower extremity surgery. Coadministration of epidural opioids and local anesthetics allows for a reduction in drug dosage for both medications, clinically similar efficacy in pain control, and a reduction in drug-related adverse effects [10]. However, patient-controlled analgesia (PCA) remains a widely used postoperative pain control modality. Chauvin et al. found that while there was a considerable reduction in drug (alfentanil) consumption in the epidural group compared to the PCA group, the incidence of oxyhemoglobin desaturation between the two groups remained similar [10-11]. Another study showed no significant differences in the incidence of drug-related complications, such as over-sedation and respiratory depression between epidural and PCA groups, but did note a modest improvement in analgesia [9]. 

Epidural analgesia is often continued for two to three days post surgery, depending on institution protocols [12]. During this time, a Foley catheter is left in place until the epidural catheter is discontinued. Due to the inability to direct neural-blockade to a single extremity, physical therapy is often delayed, resulting in longer hospital stays. While many studies have compared the effectiveness of epidural and non-epidural pain control modalities in both pediatric and adult populations for various surgical procedures, this study is the first to compare these modalities in patients with neuromuscular conditions undergoing unilateral hip reconstruction. We hypothesized that patients whose pain was controlled with non-epidural analgesia would have the same level of pain control, no differences in respiratory complications, but shorter length of stay, and shorter duration of Foley placement compared to those treated with epidural analgesia. This study sought to determine if epidural analgesia used for patients with neuromuscular conditions undergoing hip reconstruction (proximal femur osteotomies, acetabular osteotomies, or both) was non-inferior to traditional pain control regimens. Additionally, we sought to quantify differences in pain scores, duration of pain control, duration of Foley placement, and overall complication rate.

## Materials and methods

After obtaining approval from the Children's National Hospital Institutional Review Board (Pro00013336), a retrospective chart review of pediatric hip reconstructive procedures in which either epidural or non-epidural analgesia was administered for postoperative pain relief. We identified patients who underwent unilateral or bilateral proximal femoral osteotomies, pelvic osteotomies, or hip open reductions from 01/01/2009 - 12/02/2019 at Children's National Hospital, which yielded 227 patients. We excluded patients who had a revision surgery and those without neuromuscular conditions. The final cohort consisted of 70 cases that met all criteria, of which 58 underwent unilateral procedures. Demographics, laterality of surgery, postoperative pain control modality, duration of pain control modality, length of stay, and postoperative complications were recorded. Pain scores were recorded using the Face, Legs, Activity, Cry, Consolability (FLACC) pain scale. A low, high, median, and mean pain score was recorded for each patient. Complications were defined as readmission to hospital for a respiratory or orthopaedic issue including but not limited to re-dislocation or loss of surgical fixation. Additionally, we included soft-tissue complications, such as pressure ulcers, or any superficial or deep wound infection.

Summary statistics on demographic and baseline patient characteristics were presented as mean with standard deviation for continuous data and frequencies with percentages for categorical data. Continuous and categorical baseline variables were compared between epidural and non-epidural groups using unpaired t-test and Chi-square test or Fisher’s exact test (if expected cell count <5) respectively. Normality assumptions were checked using a statistical test (e.g., Shapiro Wilk test) as well as graphical methods (e.g., histogram, q-q plot). Only the length of stay variable was found to significantly deviate from normality.

Pain scores, duration of Foley placement, and duration of pain control modality between two groups were compared using unpaired t-test followed by multiple linear regression analysis adjusted for potential confounders age, BMI, procedure type, and immobilization type. Length of stay was compared using non-parametric Mann-Whitney U Test. Complication rates including hospital readmission, respiratory difficulties, re-dislocation, loss of surgical fixation, and soft-tissue complications were compared using Chi-square test. All statistical tests were two-sided and performed at the 0.05 level of significance. Statistical analysis was performed using R statistical software, version 4.0.0 (R Core Team, 2020).

## Results

Of the 70 patients in the dataset, 58 patients underwent unilateral procedures, 28 (48%) used non-epidural pain-control modalities, and 30 (52%) used epidural analgesia. Both groups were comparable in terms of the baseline characteristics except for BMI which was slightly higher in the non-epidural group (Table 1).

**Table 1:** Demographic and baseline patient characteristics SD = Standard deviation, ASA = American Society of Anesthesiologists P-values were obtained from unpaired t-test for continuous data, and Chi-square/Fisher’s exact test for categorical data

Patient characteristics	Overall	Epidural	Non-epidural	P value
	(N=58)	(N=30)	(N=28)	
Age (years), mean (SD)	9.5 (5.7)	8.8 (4.5)	10.2 (6.9)	0.358
Height (cm), mean (SD)	119.4 (24.0)	119.3 (23.4)	119.5 (25.0)	0.975
Weight (kg), mean (SD)	26.3 (11.9)	24.6 (10.2)	28.1 (13.3)	0.259
BMI (kg/m^2^), mean (SD)	17.4 (3.9)	16.0 (3.4)	18.8 (3.8)	0.006
ASA, n (%)				
Class 1	1 (2.2)	1 (4.0)	0 (0.0)	0.663
Class 2	7 (15.6)	3 (12.0)	4 (20.0)	
Class 3	36 (80.0)	21 (84.0)	15 (75.0)	
Class 4	1 (2.2)	0 (0.0)	1 (5.0)	
Immobilization Type, n (%)				
Petrie	20 (37.7)	7 (25.0)	13 (52.0)	0.078
Pillow	9 (17.0)	7 (25.0)	2 (8.0)	
Spica	24 (45.3)	14 (50.0)	10 (40.0)	
Procedure, n (%)				
Combined	37 (63.8)	16 (53.3)	21 (75.0)	0.112
Femur	13 (22.4)	10 (33.3)	3 (10.7)	
Hip	8 (13.8)	4 (13.3)	4 (14.3)	
Open Reduction, n (%)	28 (48.3)	15 (50.0)	13 (46.4)	0.993
Number of comorbidities, mean (SD)	6.3 (2.8)	6.6 (3.0)	6.0 (2.6)	0.445

Pain scores (mean, median, high, and low), length of stay, duration of Foley placement, duration of pain control modality, and complications were compared between the two groups. Average pain scores were similar between the two groups. Duration of Foley placement was significantly higher in the epidural group compared to the non-epidural group (P=0.008). Although complications were higher in the epidural group, it was not statistically significant (26.7% vs. 10.7%, P=0.121) (Table 2).

**Table 2:** Comparison of pain scores, length of stay, Foley catheter duration, pain control modality, and complications SD = Standard deviation, IQR = Interquartile Range P-values were obtained from unpaired t-test or Mann-Whitney U Test (if skewed) for continuous data, and Chi-square test for categorical data

Variables	Epidural (N=30)	Non-epidural (N=28)	P value
Pain Score Mean, mean (SD)	3.5 (1.1)	3.7 (0.8)	0.446
Pain Score Median, mean (SD)	3.5 (1.4)	3.5 (1.1)	0.881
Pain Score High, mean (SD)	6.0 (2.1)	6.4 (1.9)	0.536
Pain Score Low, mean (SD)	1.3 (0.7)	1.8 (1.2)	0.070
Length of Stay (days), median (IQR)	4.6 [3.8, 6.2]	3.3 [3.0, 6.2]	0.171
Foley Duration (days), mean (SD)	2.7 (1.0)	1.8 (1.3)	0.008
Pain Control Duration (days), mean (SD)	2.5 (1.0)	2.0 (1.9)	0.276
Complications, n (%)	8 (26.7)	3 (10.7)	0.121

We further compared these variables between two groups using multiple linear regression adjusted for age, BMI, procedure type, and immobilization type. This adjusted analysis revealed that length of stay and duration of Foley placement was significantly higher and pain score low was significantly lower in the epidural group compared to the non-epidural group (Table 3). 

**Table 3:** Multiple linear regression analysis adjusted for age, BMI, procedure type, and immobilization type CI = Confidence interval

Variables	[Epidural - Non-Epidural] (95% CI)	P value
			Pain Score Mean	-0.06 (-0.69, 0.57)	0.846
Pain Score Median	0.13 (-0.64, 0.89)	0.737
Pain Score High	0.26 (-1.11, 1.64)	0.699
Pain Score Low	-0.77 (-1.42, -0.11)	0.022
Length of Stay (days)	3.18 (0.28, 6.07)	0.032
Foley Duration (days)	1.04 (0.23, 1.84)	0.013
Pain Control Duration (days)	0.11 (-1.13, 1.35)	0.854

## Discussion

After controlling for demographic variability, our data suggest there are significant differences in the length of stay, duration of Foley placement, and the lowest reported pain score between the epidural and non-epidural groups. It is important to note, the specific medications in the epidural and non-epidural groups were not evaluated. Additionally, we did not specifically review if the non-epidural group received peripheral nerve blocks or intravenous local anesthetic infusions.

The multiple linear regression analysis showed that the epidural group had a statistically significant increase in length of stay and duration of Foley placement, and significantly lower pain score than the non-epidural group (P=0.032, P=0.013, P=0.022), but there were no significant differences in any of the other pain score parameters. These results demonstrate that the pain control is comparable between epidural and non-epidural analgesia, but that epidural analgesia has the additional drawbacks of increasing length of stay and duration of Foley placement in this patient population. It is important to note, while the difference in lowest pain score values between the groups was statistically significant, no significant conclusions can be drawn from this particular parameter, as the other pain score parameters were not statistically significant.

Patients receiving epidural analgesia maintained their Foley catheter for an average of 1.04 more days than the non-epidural patients, which could pose a greater risk of complications related to catheterization such as urinary tract infection [13]. However, in our two cohorts, there was no difference in urinary tract infection (UTI) incidence. Of note, patients receiving epidural analgesia had a length of stay that was on average 3.18 days greater than the non-epidural group. Increased length of stay is associated with a number of negative health consequences, such as increased mortality, increased risk of infection, increased risk of adverse medication side effects, and decreased quality of care [13-14]. Furthermore, several studies have shown that epidural analgesia has a high failure rate [12,15-20].

Although the difference in the number of complications between the two groups was not statistically significant, the increased number of complications in the epidural cohort is important to note. More specifically, placement of an epidural catheter has been shown to potentially further increase the risk of adverse complications, including epidural abscess, epidural hematoma, and post-dural puncture headache [18]. At the same time, none of the epidural patients included in this study experienced these described complications as a result of catheterization. Alternatively, a systematic review by Guay et al. showed that there were no conclusive differences in the risk of complications such as respiratory depression, epidural abscess, wound infection, or neurological complications between epidural and non-epidural groups in the acute setting for postoperative pain control following thoraco-lumbar spine surgery in children [15].

Prior studies have compared epidural and non-epidural PCA pain control modalities, but the literature is inconclusive on whether epidural analgesia offers any additional benefit to postoperative treatment when compared to traditional multimodal pain control regimens. Chauvin et al. demonstrated that epidural administration of alfentanil resulted in a 39% decrease in opioid consumption as compared to the non-epidural PCA modality, but ultimately, the oxyhemoglobin desaturation between the two groups was comparable [10-11].

Other studies suggest no significant differences in the incidence of drug-related complications, such as over-sedation, oxygen desaturation, and respiratory depression between epidural and PCA groups, and describe a modest improvement in analgesia [21]. However, Moore et al. and Duarte et al. showed that epidural analgesia significantly improved pain control and decreased incidence of respiratory depression compared to non-epidural analgesia [8,16].

This study does have several limitations. Primarily, it was retrospective nature, there was a lack of randomization among our sample, which may pose a selection bias. The selection bias is inherent in a retrospective review like this, as an epidural may have been placed in children who were having more extensive surgery or bilateral operation. This could also be an alternative explanation for why there were longer stays in the epidural group. Additionally, although baseline demographic characteristics were not significantly different, our sample had a wide spectrum of BMI recordings which poses a challenge in ruling out possible comorbidities influencing our findings. Furthermore, pain scores obtained from our patient sample are limited by the difficulty of attaining pain scores from children with neuromuscular conditions, such as cerebral palsy, given their inability to reliably self-report pain location and intensity.

Studies suggest that children with CP already experience twice as many complications and have high reoperation rates following hip surgery compared to their non-CP counterparts [22-23]. Therefore, to optimize outcomes in an already at-risk patient population likely to undergo multiple procedures to correct musculoskeletal abnormalities, it is crucial to minimize opioid usage given its addictive nature and side effects such as constipation, sedation, and tolerance. We believe the present study can serve as a foundation for future prospective and multi-center studies, which should aim to investigate dose and timing of epidural analgesia in children with neuromuscular conditions with a particular focus on surgical approach, side effects, and time to return to activity.

## Conclusions

Use of epidural analgesia in children with neuromuscular conditions was associated with comparable pain scores, despite the increased length of stay and increased duration of Foley placement. No statistically significant difference in complication rates was observed between patients receiving epidural anesthesia and those receiving traditional pain modalities. Given the paucity of data surrounding the use of epidural anesthesia in children with neuromuscular conditions, future research should seek to further investigate the efficacy of epidural analgesia for postoperative pain management in children with neuromuscular conditions.
